# Photosensitive Alternative Splicing of the Circadian Clock Gene *timeless* Is Population Specific in a Cold-Adapted Fly, *Drosophila montana*

**DOI:** 10.1534/g3.118.200050

**Published:** 2018-02-22

**Authors:** Riikka Tapanainen, Darren J. Parker, Maaria Kankare

**Affiliations:** * University of Jyväskylä, Department of Biological and Environmental Science, FI-40014 Jyväskylä, Finland and; † Department of Ecology and Evolution, University of Lausanne, Biophore, Lausanne 1015, Switzerland

**Keywords:** Alternative splicing, *Drosophila montana*, light-dark cycle, *timeless*, temperature

## Abstract

To function properly, organisms must adjust their physiology, behavior and metabolism in response to a suite of varying environmental conditions. One of the central regulators of these changes is organisms’ internal circadian clock, and recent evidence has suggested that the clock genes are also important in the regulation of seasonal adjustments. In particular, thermosensitive splicing of the core clock gene *timeless* in a cosmopolitan fly, *Drosophila melanogaster*, has implicated this gene to be involved in thermal adaptation. To further investigate this link we examined the splicing of *timeless* in a northern malt fly species, *Drosophila montana*, which can withstand much colder climatic conditions than its southern relative. We studied northern and southern populations from two different continents (North America and Europe) to find out whether and how the splicing of this gene varies in response to different temperatures and day lengths. Interestingly, we found that the expression of *timeless* splice variants was sensitive to differences in light conditions, and while the flies of all study populations showed a change in the usage of splice variants in constant light compared to LD 22:2, the direction of the shift varied between populations. Overall, our findings suggest that the splicing of *timeless* in northern *Drosophila montana* flies is photosensitive, rather than thermosensitive and highlights the value of studying multiple species and populations in order to gain perspective on the generality of gene function changes in different kinds of environmental conditions.

Adaptation to environmental changes *e.g*., in temperature and day length, is crucial for the survival of most organisms. While insects’ responses to cold temperatures can be highly variable, they usually involve changes in reproduction or diapause strategies and metabolism, as well as in gene expression linked with these traits (*e.g.*, Thieringer *et al.* 1998; Hoffmann *et al.* 2003; Vesala *et al.* 2012a, b; Parker *et al.* 2015). However, gene expression studies typically overlook changes at the transcript level, such as differential variant usage resulting from alternative splicing (AS). Because of the overwhelming prevalence of AS forms across the genome of many Eukaryotes and across different functional classes of genes (Irimia *et al.* 2007), it is very likely that the alternatively spliced variants of central genes influence the way in which organisms adjust to environmental change.

To maximize their fitness, organisms have to adjust their physiology and behavior in response to a suite of varying environmental conditions. Central to the regulation of these changes is an organism’s internal circadian clock (Takahashi and Zatz 1982; Boothroyd *et al.* 2007; Zheng and Sehgal 2008), and recent evidence has suggested that clock genes are important also in the regulation of seasonal adjustments (Montelli *et al.* 2015; Denlinger *et al.* 2017). Regulation of the circadian clock itself has been well described in *Drosophila melanogaster*, where both the temperature and the photoperiod have been found to alter the expression of two core clock genes, *timeless* (*tim*) and *period* (*per*) in fly heads (Boothroyd *et al.* 2007). In light-entrainment, the expression of these genes is tightly coupled, but the coupling breaks down in temperature-entrainment, which leads to an advance in the expression of *per* and delay in the expression of *tim*, but induces no differences at protein level (Boothroyd *et al.* 2007). There are several potential explanations on how the equal accumulation of PER and TIM proteins could be maintained regardless of the different expression levels. First, there could be a shift in the timing of the expression of both genes (Boothroyd *et al.* 2007) or the advance in the expression of *per* could be caused by thermosensitive splicing of this gene, which would then effect the expression levels of *tim* (Majercak *et al.* 1999; Collins *et al.* 2004). Finally, splicing occurring in both of these genes could explain the difference in their expression levels (Boothroyd *et al.* 2007). Studies on *D. melanogaster* have shown that *tim* has two different transcript variants that are thermosensitively spliced (Boothroyd *et al.* 2007; Montelli *et al.* 2015): *tim^spliced^* and *tim^unspliced^*. The latter one is a longer transcript, in which the last intron is retained causing a premature stop codon and resulting in the production of a truncated protein (the amino acids encoded by the last exon are missing (Boothroyd *et al.* 2007)). This truncated protein lacks part of the cytoplasmic localization domain, which may contribute to fine-tuning of PER and TIM protein oscillations with the daily thermal cycle (Boothroyd *et al.* 2007). It is also known that the longer variant (*tim^unspliced^*) in *D. melanogaster* flies has higher expression levels at low temperature (18°) than at high temperature (25°) (Boothroyd *et al.* 2007). Montelli *et al.* (2015) also investigated different splicing variants of *tim* in natural conditions throughout the seasons. They found that *tim^spliced^* expression is increasing at higher temperatures and also that the total amount of *tim* mRNA expressed is influenced by seasonal and even daily changes of temperature and day length in the wild (Montelli *et al.* 2015).

Interestingly, in a northern, highly cold tolerant *D. montana* fly, peak expression levels of *tim* and *per* genes have been found to be similar to each other in several conditions from continuous light in early summer to the shorter day lengths and cooler temperatures in late summer, typical to northern environments (Kauranen *et al.* 2016). Consequently, both the photoperiod and the daily temperature cycles are important cues for seasonal changes in the circadian rhythm of *D. montana* (Kauranen *et al.* 2016). This offers a good possibility to the study fundamentals of expression level differences, including patterns of AS, in *tim* and to get some insights on their role in adaptation to seasonally varying environmental conditions. In the current study, we collected information from the splicing variants of *tim* in *D. montana* flies from two temperature and photoperiodic conditions. We also examined the effects of these conditions on the expression levels of *tim* variants in northern and southern populations from two continents. Alternative splicing is an important genetic mechanism that may also help species to adapt to different environments (*e.g.*, Jakšić and Schlötterer 2016) and information on specific clock genes and their splicing patterns could be of the utmost importance to understand these patterns.

## MATERIAL AND METHODS

### Fly strains and rearing

**Figure 1:**
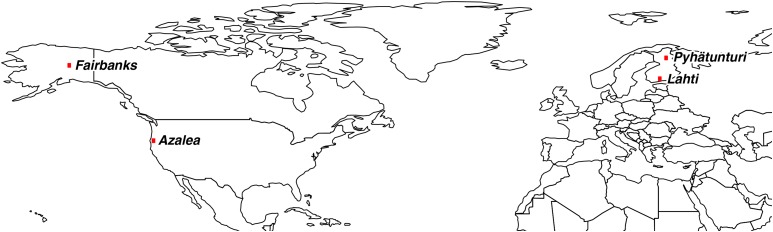
*D*. *montana* fly populations used in the study. Fly collection sites in Fairbanks (Alaska, northern population) and Azalea (Oregon, southern population) in North America, with geographical distance between the collection sites more than 4000 km. Finnish flies originated from Pyhätunturi (northern population) and Lahti (southern population) with a distance between the sites approximately 700 km.

We used isofemale strains of *D. montana* established from the progenies of mated females collected in the wild from two extremes of a latitudinal cline in the USA (Fairbanks, Alaska; 64°55’N and Azalea, Oregon; 42°48’N) and in Finland (Pyhätunturi; 67°06’N and Lahti; 60°59’N) (Figure 1, Supplementary material, Table S1 in File S1). The flies were collected using malt traps near small rivers and streams and reared in 250 ml bottles containing malt medium (Lakovaara 1969) in constant light (LL 24:0) and at 19° since their establishment. For the current experiment, ten randomly chosen females and ten males from each of the studied strains were moved into new 250 ml malt medium bottles and transferred into a maintenance chamber with two temperature and two lighting conditions (*i.e.*, we had 4 replicate bottles from each strain). After the flies had mated and laid eggs, the founder flies were removed from the bottles to ensure that the flies (progeny) used in this study had been reared in the correct conditions from egg to adult. Half of the flies were reared in constant light (LL) and the other half in day length of 22 hr of light and two hours of darkness (LD 22:2). Both of these light regimes prevent the flies of all our study populations from entering diapause, which could cause differences in the expression level of many circadian clock genes (Salminen *et al.* 2015). In both lighting conditions, the flies were further divided into two temperature groups: 19° and 16°. The higher temperature was the same where the original isofemale strains had been maintained since their establishment and the lower temperature used was 16°. Both of these temperatures resemble those in flies’ natural sites during the summer months except the temperatures from southern Azalea which would be too high for northern flies and already cause thermal acclimation (See Supplementary Table S8 in File S1). We collected two females randomly from each isofemale strain and used three strains for constant light and two strains for LD 22:2 from each population (Supplementary Table S1 in File S1). Flies were collected at the same time of the day at 1.00 pm (13:00) from both light dark-cycles at the age of 2-3 weeks (*i.e.*, when they were sexually mature) quickly frozen with liquid nitrogen, and stored at -84° prior to RNA extraction. The dark period of 2 hr in LD 22:2 was always at 00.00-02.00 am.

### RNA extractions, cDNA synthesis and molecular cloning of the different transcripts

**Figure 2:**
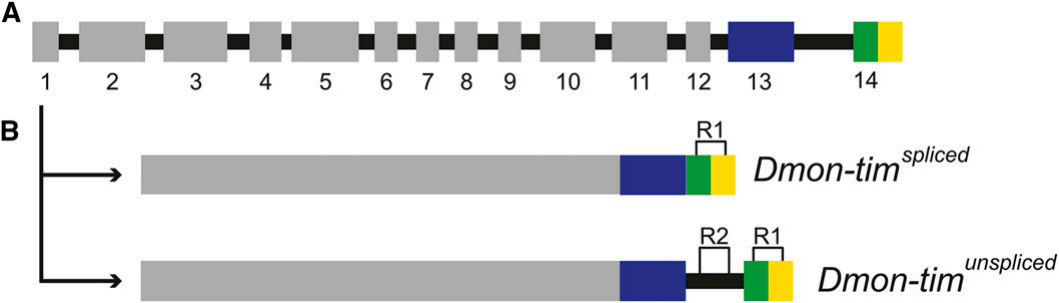
*timeless* gene in *D. montana*. A) Schematic presentation of *timeless* gene in *D. montana* showing exons 1-12 (gray), exon 13 (blue), exon 14 (green), UTR (yellow), and introns (black). Note that introns are not in scale. B) Schematic presentation of the two splice variants, *Dmon-tim^spliced^* and *Dmon-tim^unspliced^*. In the first variant the intron between exons 13 and 14 has been spliced out, whereas in the second variant it has been retained. R1 and R2 show the regions of the transcripts amplified in the qPCR (see methods for details).

RNA was extracted from whole female flies with Direct-zol RNA MiniPrep Kit (Zymo Research) with DNAse treatment, and the purity of the RNA was measured with a NanoDrop ND-1000 (Thermo Scientific) using ND-1000 V3.8.1 software. RNA concentration of each sample was measured with Qubit 2.0 Fluorometer (Invitrogen, Thermo Fisher Scientific) using Qubit RNA HS Assay Kit. cDNA synthesis was carried out with SuperScript III First-Strand Synthesis System for RT-PCR (Invitrogen, Thermo Fisher Scientific) using equal amounts (100 ng) of RNA. The 3′ end of the gene (from exon 13 until beginning of 3′UTR region, Figure 2) was amplified from the cDNA using PCR and tim_ex13_1F and tim_3′UTR_Rb primers (Supplementary Table S2 in File S1) and following protocol: 95° for 1 min, 95° for 15 s, 55° for 15 s, 72° for 20 s, 20° for 2 min. Molecular cloning was used to investigate the presence of different transcripts in different samples. The cloning was carried out using a CloneJET PCR Cloning Kit (Fermentas, Thermo Fisher Scientific) using manufacturer’s instructions and LuriaBroth ampicillin plates. PCR products were ligated into the vector (pJET1.2/blunt Cloning vector) and transformed into *E. coli* Zymo 5 alfa (Zymo Research, T3007) cells. Plates were incubated at 37° overnight (14-16 hr) and 70 colonies from each sample were picked from the plates. From 1680 collected colonies, 228 were randomly chosen to be amplified with PCR using pJET1.2 F and R primers (Supplementary Table S2 in File S1) and run in agarose gels to check which of them had the expected transcript lengths (about 500 and 1000 bp). A total of 19 products with expected lengths were then chosen randomly to be sequenced with ABI 3130xl Genetic Analyzer (Thermo Fisher Scientific) using pJET1.2 F and R primers. Sequenced products were analyzed using Sequence Analysis software (Thermo Fisher Scientific) and then aligned using PRANK (v.100802, Löytynoja and Goldman 2005) with default options.

### Quantitative real time PCR (qPCR)

Differences between the expression levels of the two splice variants of *timeless* were measured with qPCR using the above-mentioned cDNA samples and tim_intron_13_2FR, tim_exon_14F and tim_3′UTR_Rb primers (Figure 2, Supplementary Table S2 in File S1) with Bio-Rad’s CFX96 Real-Time System C1000 Touch thermal cycler. qPCR protocol used was: 95° for 3 min, 95° for 10 s, 55° for 10 s, 72° for 30 s, 95° for 10 s and finally melting curve analysis. The gene expression levels were calculated with normalized expression method (∆∆(Ct)) (Livak and Schmittgen 2001) using real efficiency values for the primers (Supplementary Table S3 in File S1) and CFX Manager v. 3.1 (BioRad). *Ribosomal protein L23* (*RpL32*) and *18S Ribosomal RNA* (*18S*), which showed equal expression levels in all samples, were used as control genes. Each run included 3 technical replicates for each sample, and the final threshold value (Cq) was defined as a mean of the technical replicates that produced good quality data. The primers used in qPCR bind either to intron 13 or to exon 14 and the 3′UTR region (Figure 2, Supplementary Figure S1 in File S1). The primer pair that binds only to intron 13 tracks the amount of expression of the longer variant (intron 13 included, *Dmon-tim^unspliced^* see Figure 2), whereas the primer pair that binds to exon 14 and in 3′UTR tracks the expression levels of both variants, as both of them include exon 14 at the transcriptional level (*Dmon-tim^unspliced^* and *Dmon-tim^spliced^* , Figure 2).

### Statistical analyses

The expression values collected from the qPCR runs were used to calculate the ratio of expression levels between the intron-containing long transcript (*Dmon-tim^unspliced^*) and both transcripts containing the exon region (hereby referred to as the long/both variant(s) ratio), for each sample. A type III 3-way ANOVA implemented in R (version 3.1.2) (R Core Team 2016) using the car package (Fox and Weisberg 2011) was used to trace the effects of temperature and lighting treatments and population, as well as possible interactions between them, on the long/both variant(s) ratio in our samples. The assumptions of ANOVA were met, as the data were normally distributed (normality of the dependent variable (long/both variant(s) ratio) was checked with Shapiro–Wilk test (Royston 1995) and the samples were independent. After ANOVA, Tukey’s HSD test (Tukey’s honest significant difference) (Miller 1981) was used to examine pairwise differences between the groups.

### Data availability

All generated sequences have been deposited into GenBank under the following accession numbers: MG279509 - MG279527.

Supplementary Material includes Tables S1–S8 and Figures S1 and S2 in File S1.

File S2 contains the full alignment of the sequenced areas of *timeless* gene.

## RESULTS

### Different *tim* splicing variants in *D. montana*.

Sequencing of the cDNA collected from two different temperature and light-dark (LD) treatments identified two different splicing variants, *Dmon-tim*^spliced^ and *Dmon-tim*^unspliced^, with the product lengths of 284 and 800 nucleotides (Figure 2, Supplementary Figure S2 in File S1). The length difference was caused by the retention of the intron between exons 13 and 14 in the longer transcript. Before sequencing, the variants were cloned to separate the long and short transcripts from each other and run on agarose gel electrophoresis to identify their length in each picked colony. The difference between the product lengths was 516 bp, which caused both the PCR and cloning to be biased toward the shorter transcript (less than 1/10 of the colonies had the longer product).

**Figure 3:**
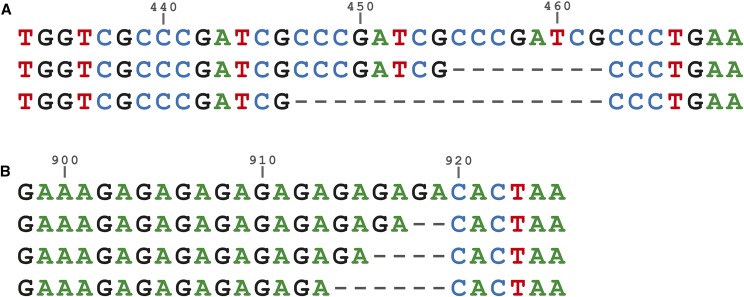
Repeat variation in *timeless* intron 13 and 3′UTR. Sequence alignment produced by PRANK showing the length differences in the *timeless* gene, caused by repeat regions. Numbers indicate base position in full alignment provided in Supplementary Data S1. A) Repeat region of CCCGATCG in the middle of intron 13 and B) dinucleotide repeat of GA in the 3′UTR region.

In addition to the two expected splice variants, we also found a few variants with unexpected sizes. Up to 16 nucleotide differences in the lengths of these variant were caused by insertions/deletions of the “CCCGATCG” repeat region in the middle of the intron 13 (Figure 3A). Moreover, at the 3′UTR, a dinucleotide region of GA repeated 6-9 times (Figure 3B) was found from all four fly populations from Finland and USA (for the full alignment see File S2). We did not find any correlation between the number of repeats and particular population, *i.e.*, none of the repeat types were restricted to a single population.

### Gene expression levels of different *tim* variants

**Figure 4:**
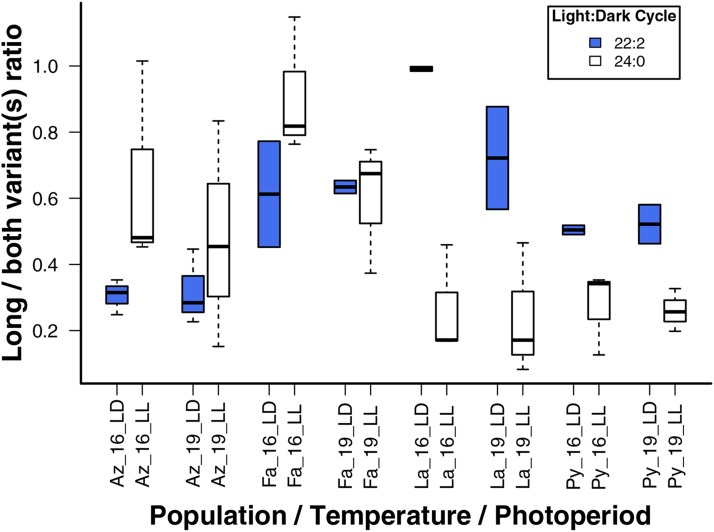
Comparison of long/both variant(s) normalized expression ratio of *timeless* in different populations from different light-dark cycles and temperatures. Conditions are given in the form: population, temperature (°C), and light dark cycle separated by an underscore. Populations are abbreviated as: Az = Azalea, Fa = Fairbanks, La = Lahti and Py = Pyhätunturi. Blue boxes indicates the LD of 22:2 and white boxes constant light (LL).

Expression level analysis involved two primer pairs, the first of which binds to intron 13 and the second one to exon 14 and 3′UTR region (Supplementary Figure S1 in File S1). The first pair of primers gave information on the expression level of the longer splice variant (*Dmon-tim*^unspliced^), including the intron sequence, and the second pair on the expression levels of both products (as both variants include the exon 14). These expression levels were then used to calculate the long/both variant(s) ratio in different fly samples. An ANOVA comparing these ratios between all populations and treatments detected three statistically significant factors, including the effect of population (*P* < 0.001), light treatment (*P* < 0.05) and the interaction between them (*P* < 0.001), while temperature did not have a significant effect (*P* > 0.90) (Table 1). Interestingly, significant interaction between population and light treatment seems to be driven by the fact that in the USA populations (Fairbanks and Azalea) the long/both variant(s) ratio is higher in constant light (LL) than in LD 22:2, while in the Finnish populations (Lahti and Pyhätunturi) the situation is the opposite (Figure 4).

**Table 1:** The ANOVA analysis comparing long/both variant(s) ratios of *timeless* among all the populations and treatments. The effect of temperature, population and light treatment assessed by ANOVA (full model). Abbreviations: Temp = temperature, Pop = population and Light = light treatment. Significant p-values are bolded. Total number of replicates = 42

**Treatment**	**Sum of Sq**	**Df**	**F value**	**ω^2^**	**P**
(Intercept)	0.2798	1	7.9440	0.078	0.0091**
Temp	0.0003	1	0.0079	−0.011	0.9300
Pop	0.5772	3	5.4623	0.150	0.0048**
Light	0.1777	1	5.0458	0.045	0.0334*
Temp*pop	0.0639	3	0.6044	−0.013	0.6181
Temp*light	0.0252	1	0.7150	−0.003	0.4055
Pop*light	0.9642	3	9.1245	0.273	0.0003***
Temp*pop*light	0.1105	3	1.0460	0.002	0.3888
Residuals	0.9158	26			

Significance levels: * *P* < 0.05, ** *P* < 0.01, *** *P* < 0.001.

Since a number of the higher-order terms in the full ANOVA model were non-significant, a reduced model was produced by sequentially dropping the non-significant highest-order term with the highest p-value. Following this procedure the reduced model consisted of the following terms: temperature, light treatment, population, and the interaction between population and light treatment (Supplementary Table S4 in File S1). Results from the reduced model were similar to the results obtained from the full model, with population (*P* < 0.001), light treatment (*P* < 0.05) and interaction between of population and light treatment (*P* < 0.001) showing significant effects. Moreover, examination of pairwise differences between the treatment groups for temperature, light and population revealed several significant differences, most of which were between the two Finnish populations (Supplementary Table S5 in File S1, for statistically significant results of pairwise comparisons see Supplementary Table S6 in File S1 and for all pairwise comparisons see Supplementary Table S7 in File S1).

## DISCUSSION

Alternative splicing of genes is an important genetic mechanism, as it provides an additional source of variation for selection at the genetic level, which may be crucial for the species’ adaptation into seasonally changing environments. In many insect species the circadian clock, which regulates many changes in physiological and behavioral traits, is entrained by rhythmic changes in light and temperature cues (Montelli *et al.* 2015; Denlinger *et al.* 2017). Here, we investigated the splicing patterns of a core circadian clock gene, *timeless* (*tim*), in northern and southern *D. montana* populations from two different continents (Europe and North America). Overall, our findings suggest that the splicing of *tim* in northern *D. montana* flies is photosensitive, rather than thermosensitive contrary to the situation in the widespread cosmopolitan species, *D. melanogaster* (Boothroyd *et al.* 2007; Montelli *et al.* 2015). Interestingly, we also found that a change in the photoperiod induced a shift in the relative abundances of *tim* variants on both continents, but in different directions. Moreover, in Finnish populations the level of splicing was similar in constant light but not in LD 22:2, while in North America it showed different patterns in both photoperiods. This highlights how idiosyncratic genetic changes can be even within a single species and the value of using multiple species and populations in these type of studies.

The circadian clock is the main time-keeping mechanism in a wide range of organisms. When investigating the splicing of the core circadian clock genes, like *tim*, it is important to take into account that the role and the function of the clock can vary between the species. Importantly, the northern *D. montana* flies are known to be able to maintain their free-running locomotor activity rhythm in constant light but not in constant darkness, opposite to the behavior of more southern species like *D. melanogaster* (Kauranen *et al.* 2012) and the same pattern was also found from two other northern *D. virilis* group fly species *D. ezoana* and *D. littoralis* (Menegazzi *et al.* 2017). This is suggested to be an adaptation to the long (even continuous) days during the flies’ mating season in early summer in the northern latitudes (Kauranen *et al.* 2012). Kauranen *et al.* (2016) also investigated locomotor activity of *D. montana* flies in different light-dark cycles for two weeks and found out that in LL (19°) the flies free run with period of 23 hr and in LD 22:2 (19/13°) the flies entrained rhythms were very close to 24 hr. The authors also resolved that the mean locomotor activity did not change between the groups of flies collected from the above mentioned light dark cycles (Kauranen *et al.* 2016). Moreover, *D. montana* lacks the morning activity peak typical to *D. melanogaster* (Kauranen *et al.* 2012) as do also *D. ezoana* and *D. littoralis* (Menegazzi *et al.* 2017). All of these differences could induce the differences detected in the splicing patterns of *tim* in above-mentioned species, but the splicing frequency of the *tim* can also be influenced by the expression of the other core circadian clock genes, including *per*. In *D. montana*, the expression levels of *tim* and *per* coincide in the same light-dark cycles and temperatures (Kauranen *et al.* 2016), while in *D. melanogaster* in light-entrainment, the expression of these genes is tightly coupled, but the coupling breaks down in temperature-entrainment leading to an advance in the expression of *per* and delay in the expression of *tim* (Boothroyd *et al.* 2007).

In natural conditions, flies need to respond to variation in light and temperature at both daily and seasonal scales. Among the photoperiods used in this study, constant light (24:0) represents situation in early summer at high latitudes (Supplementary Table S8 in File S1) and in this lighting condition the circadian rhythms of the flies are free-running but the flies still retain their rhythmicity (Kauranen *et al.* 2016). The second photoperiod, light-dark cycle of LD 22:2, represents mid-summer at high latitudes (Supplementary Table S8 in File S1), and here the circadian rhythms are entrained by the two hours dark period. These photoperiods prevent both the northern and the southern flies from entering diapause, which is important for the scope of the present study (Salminen *et al.* 2015). Although a difference of two hours between the light cycles may appear small, it in fact may represent a large difference in the environmental conditions for different populations of northern-distributed fly such as *D. montana*. For example, day length increases by 4.8 hr in northern Pyhätunturi (Finland) and by 2.5 hr in Fairbanks (USA) populations from May to June, while in the southern Lahti (Finland) and Azalea (USA) populations the change is only 1.7 and 1.0 hr, respectively (Supplementary Table S8 in File S1). Hence, for the more northern populations a 2 hr difference in LD equates to a short time period (*i.e.*, only 2 weeks) during midsummer while for more southern species it means a longer time period (up to 2 months) when other environmental conditions such as temperature may have changed greatly. Unexpectedly, the direction of a change induced by the photoperiod (constant light *vs.* LD 22:2) on *tim* splicing differed between the USA and Finnish populations. In the USA populations the expression of the longer variant (*Dmon*-tim^unspliced^) was higher in constant light (LL) than in LD of 22:2, while in the Finnish populations the pattern was the opposite. One thing that may have affected our results is that, although Finnish flies from both of the used populations and USA flies from northern Fairbanks experience very long day lengths during the summer in the wild, constant light is natural only for the Pyhätunturi population in June (see Supplementary Table S8 in File S1). The effect of very long and unnatural light conditions in the splicing of *tim* is not known but it might explain the observed differences between North American and Finnish populations, as 15 hr of light is the longest photoperiod that the southern Azalea flies from USA ever experience in natural conditions while in Lahti (Finland) it is almost 19 hr of light (Supplementary Table S8 in File S1).

In addition to the thermosensitive variants at the 3′end of the *timeless* discussed above, *D. melanogaster* flies have two other *tim* variants called *ls-tim* and *s-tim*. Among these variants *ls-tim* has two translation starting points, which create long and short variants differing by 23 amino acids in the first exon, whereas *s-tim* only codes for the short product (Sandrelli *et al.* 2007; Tauber *et al.* 2007). *s-tim* appears to be the ancestral variant present in northern Europe, while *ls-tim* is a newer variant that has spread through Europe (Tauber *et al.* 2007). *ls-tim* makes the circadian clock in *D. melanogaster* less light-sensitive, which is thought to be favorable in colder climates and in environments with more variable photoperiods (Pittendrigh *et al.* 1991; Pegoraro *et al.* 2017). Montelli *et al.* (2015) investigated the affinity of 4 different TIM protein isoforms (combination of S-TIM/L-TIM and TIM^spliced^/TIM^unspliced^) and found that the light response may be stronger for *s-tim* flies under colder conditions. Interestingly, the earlier translation start site and *ls-tim* variant has not been found in *D. montana*, so these northern flies appear to only have *s-tim* variants and with different 3′end splicing variants they seem to be strongly photosensitive and well adapted to northern environmental conditions. We also want to point out that in this study we investigated the alternative splicing patterns of *tim* using whole flies, while studies with *D. melanogaster* have been mainly using the heads of the flies (Sandrelli *et al.* 2007; Montelli *et al.* 2015), which could indeed cause some differences in *tim* expression patterns between the species. However, although the expression of *tim* is known to primarily occur in the head of the flies (Myers *et al.* 1996), it is also expressed in other tissues like digestive system, fat body and ovaries of the flies (Graveley *et al.* 2011) and currently very little is known about the expression or splicing patterns of *tim* or other circadian clock genes in *D. montana* in specific tissues like head (but see Kauranen *et al.* 2016). Consequently, to get an overall idea of the splicing patterns occurring in different populations of *D. montana* from several different environmental conditions, we used whole flies and aim to move to more specific tissues in future studies.

Interestingly, we also found length differences in the longer variant from all four fly populations caused by insertions/deletions of the “CCCGATCG” repeat region in the middle of the intron 13 as well as from a dinucleotide region of GA repeated 6-9 times 3′UTR region. There was no correlation between the number of repeats and particular population, *i.e.*, none of the repeat types were restricted to a single population. The function of these repeat regions and the reason for the variability of the repeat length is currently unknown, though they might have some regulatory functions and affect *e.g.*, the expression levels of the *tim*. This could be investigated in detail in future studies as the other core circadian clock gene, *per*, is known to have thermosensitive splicing specifically in the 3′UTR region in *D. melanogaster* (Majercak *et al.* 1999).

## Conclusions

In this study we investigated the alternative splicing of an important core circadian clock gene *timeless* in several *Drosophila montana* populations in response to different temperatures and day lengths. We found that the expression of different splice variants of *tim* were not thermosensitive, but photosensitive, in contrast to earlier studies in *D. melanogaster*. We also found significant differences between the populations on two continents, as the flies from the North America (especially the southern population) expressed the longer, unspliced transcript more in constant light than in specific light-dark cycle, while flies from the Finnish populations showed the opposite expression pattern. Consequently, our results suggest that the splicing patterns of *tim* in different *D. montana* populations from different geographical origins are different, highlighting the importance of using multiple populations in order to gain perspective on the generality of gene function changes in different kinds of environmental conditions. Obviously, more studies with bigger temperature differences, a wide range of photoperiods and even more widely distributed populations are needed to better understand the presence of different variants of *tim* and their possible effects on the circadian clock rhythms of these flies in tough northern environments. Moreover, functional genetic studies with *e.g.*, RNAi and CRISPR/Cas9 gene editing systems enable to experientially manipulate the expression of different *tim* variants in multiple genetic backgrounds to determine if different splicing patterns are able to produce similar phenotypic effects in different photoperiods.

## Supplementary Material

Supplemental Material is available online at www.g3journal.org/lookup/suppl/doi:10.1534/g3.118.200050/-/DC1.
