# Supplementary material for: Photosensitive Alternative Splicing of the Circadian Clock Gene timeless Is Population Specific in a Cold-Adapted Fly, Drosophila montana
Source: G3 (Bethesda). 2018 Feb 22;8(4):1291–7. doi: 10.1534/g3.118.200050 (PMC5873918; doi:10.1534/g3.118.200050)
Supplement: Supplementary file 1 [file 1291FileS1.docx]

**Supplementary material**

**Table S1. *Drosophila montana* populations and isofemale strains used in the study**. Table gives the populations, isofemale strains, their altitudes and collections years and light-dark cycles used.

| Country | Population | Isofemale strain | Altitude (m) | Collection year | Light treatment |
| --- | --- | --- | --- | --- | --- |
| USA | Fairbanks | FA13F2 | 180 | 2013 | LL/LD |
|  |  | FA13F3 |  |  | LL |
|  |  | FA13F6 |  |  | LL |
|  |  | FA13F9 |  |  | LD |
|  | Azalea | AZA1 | 498 | 2010 | LL/LD |
|  |  | AZA2 |  |  | LL/LD |
|  |  | AZA3 |  |  | LL/LD |
| Finland | Pyhätunturi | 2PT09 | 178 | 2009 | LL/LD |
|  |  | 9PT09 |  |  | LL |
|  |  | 14PT09 |  |  | LD |
|  |  | 21PT09 |  |  | LL |
|  | Lahti | L209 | 82 | 2009 | LL |
|  |  | L409 |  |  | LL/LD |
|  |  | L509 |  |  | LL |
|  |  | L909 |  |  | LD |

**Table S2. Primer sequences used in the molecular cloning and Sanger sequencing protocols.**

| Gene | Primer sequence |
| --- | --- |
| tim_ex13_1F | 5'-CATCAGCTCCCACACAATGAC-3' |
| tim_3’UTR_Rb | 5'-AGACAATGAACCGACCCAAG-3' |
| pJET1.2 F | 5'-CGACTCACTATAGGGAGAGCGGC-3' |
| pJET1.2 R | 5'-CGACTCACTATAGGGAGAGCGGC-3' |

**Table S3. qPCR primers.** Primer sequences and their efficiency values (E%).

| Primer ID | Primer sequence F/R | E% | |
| --- | --- | --- | --- |
| tim_exon_14F | 5'-TGTCAGCGATGAGGATGAGA-3' | 90.2 | |
| tim_3'UTR_Rb | 5'-CTTGGGTCGGTTCATTGTCT-3' |  | |
|  |  |  | |
| tim_intron_13_2 | 5'-TTATGGCGGGCAATTATTCAAT-3' | 101.1 | |
|  | 5'-TGCTGCGATTAGAATTGAAGATGT-3' | | |
|  |  | | |
| 18S | 5'-AATGCACCGAGGAGGAGGTTGA-3' | | 97.6 |
|  | 5'-CGACCTGTAGTTTTGGTGTGCTGG-3' | |  |
|  |  | |  |
| RpL32 | 5'-ATCAGCAGCACCTCCAGTTC-3' | | 98.4 |
|  | 5'-GATATGCCAAGCTGTCGCACAA-3' | |  |


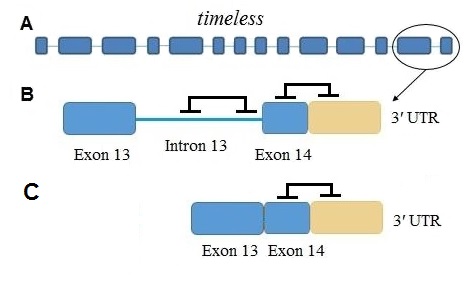


**Figure S1.** ***Drosophila montana* *timeless* gene.** A) Schematic presentation of *timeless* gene in *D. montana* (introns not in scale). (B, C) Presentation of the two splice variants used in the qPCR study (exons and intron presented in scale). Blue boxes represent exons, blue lines (connecting exons) represent introns and light brown box represents the 3′UTR area. B) Variant 1: Black horizontal lines above the intron are binding sites of tim_intron_13_2 (F & R) primers (i.e. “the intron area”). The black horizontal line above exon 14 is the binding site of tim_exon_14F primer and the line above 3′UTR is the binding site of tim_3'UTR_Rb primer (i.e. the exon area). C) Variant 2: The black horizontal lines above exon 14 and 3′UTR show the binding sites of primers and a line connecting them the whole length of the PCR product (like in B).


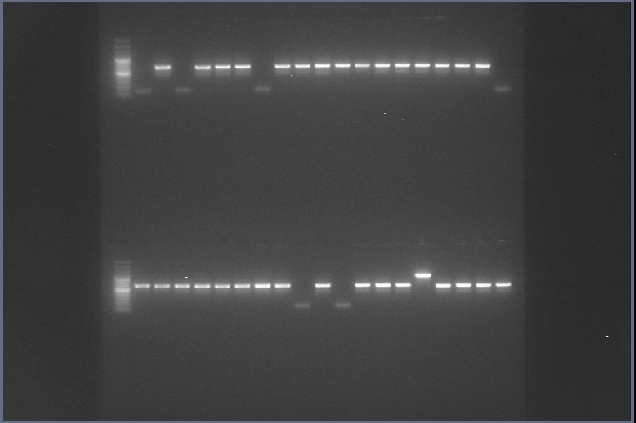


**Figure S2.** **Agarose gel electrophoresis showing the cloned PCR products.** Gel figure of the two *timeless* splice variants. Only one of the colons had the long product (marked with red circle). The lower bright band of the size standard is 500 bp and faint small bands are colons with no product.

**Table S4.** **The ANOVA analysis comparing long/both variant(s) ratios of *timeless* among all the populations and treatments.** The effect of temperature, population and light treatment assessed by ANOVA after model simplification. Abbreviations: Temp = temperature, Pop = population and Light = light treatment. Statistically significant p-values are bolded.

| **Treatment** | **Sum of Sq** | **Df** | **F value** | **P** |
| --- | --- | --- | --- | --- |
| (Intercept) | 0.6782 | 1 | 20.6095 | **0.0001***** |
| Temp | 0.0936 | 1 | 2.8447 | 0.1011 |
| Pop | 0.7419 | 3 | 7.5154 | **0.0006***** |
| Light | 0.1914 | 1 | 5.8167 | **0.0216*** |
| Pop*light | 1.1606 | 3 | 11.7567 | **0.0000***** |
| Residuals | 1.0859 | 33 |  |  |

Significance levels: * p<0.05, ** p<0.01, *** p<0.001.

**Table S5**. **Comparison of the different light treatments in different populations with Tukey's HSD test.** Abbreviations: Diff = mean difference between comparisons, Lwr = lower bound of 95% CI, Upr = upper bound of 95% CI, P adj = p-value, LL constant light, LD light-dark cycle of 22:2. Populations are abbreviated as: Az = Azalea, Fa = Fairbanks, La = Lahti and Py = Pyhätunturi. Statistically significant p-values are bolded.

| **Treatment groups compared** | **Diff** | **Lwr** | **Upr** | **P adj** |
| --- | --- | --- | --- | --- |
| Fa:LD-Az:LD | 0.3111 | -0.0675 | 0.6896 | 0.1723 |
| La:LD-Az:LD | 0.5443 | 0.1658 | 0.9229 | **0.0012**** |
| Py:LD-Az:LD | 0.2008 | -0.1777 | 0.5793 | 0.6780 |
| Az:LL-Az:LD | 0.2526 | -0.0860 | 0.5912 | 0.2691 |
| Fa:LL-Az:LD | 0.4420 | 0.1034 | 0.7805 | **0.0040**** |
| La:LL-Az:LD | -0.0594 | -0.3980 | 0.2792 | 0.9991 |
| Py:LL-Az:LD | -0.0450 | -0.3836 | 0.2935 | 0.9998 |
| La:LD-Fa:LD | 0.2333 | -0.1814 | 0.6479 | 0.6126 |
| Py:LD-Fa:LD | -0.1103 | -0.5249 | 0.3044 | 0.9877 |
| Az:LL-Fa:LD | -0.0585 | -0.4370 | 0.3201 | 0.9996 |
| Fa:LL-Fa:LD | 0.1309 | -0.2476 | 0.5094 | 0.9482 |
| La:LL-Fa:LD | -0.3704 | -0.7490 | 0.0081 | 0.0587 |
| Py:LL-Fa:LD | -0.3561 | -0.7346 | 0.0224 | 0.0774 |
| Py:LD-La:LD | -0.3435 | -0.7582 | 0.0711 | 0.1652 |
| Az:LL-La:LD | -0.2917 | -0.6703 | 0.0868 | 0.2342 |
| Fa:LL-La:LD | -0.1024 | -0.4809 | 0.2762 | 0.9864 |
| La:LL-La:LD | -0.6037 | -0.9823 | -0.2252 | **0.0003***** |
| Py:LL-La:LD | -0.5894 | -0.9679 | -0.2108 | **0.0004***** |
| Az:LL-Py:LD | 0.0518 | -0.3267 | 0.4303 | 0.9998 |
| Fa:LL-Py:LD | 0.2412 | -0.1374 | 0.6197 | 0.4601 |
| La:LL-Py:LD | -0.2602 | -0.6387 | 0.1184 | 0.3653 |
| Py:LL-Py:LD | -0.2458 | -0.6244 | 0.1327 | 0.4360 |
| Fa:LL-Az:LL | 0.1894 | -0.1492 | 0.5279 | 0.6193 |
| La:LL-Az:LL | -0.3120 | -0.6506 | 0.0266 | 0.0888 |
| Py:LL-Az:LL | -0.2976 | -0.6362 | 0.0409 | 0.1188 |
| La:LL-Fa:LL | -0.5013 | -0.8399 | -0.1628 | **0.0008***** |
| Py:LL-Fa:LL | -0.4870 | -0.8256 | -0.1484 | **0.0012**** |
| Py:LL-La:LL | 0.0143 | -0.3242 | 0.3529 | 1.0000 |

Significance levels: * p<0.05, ** p<0.01, *** p<0.001.

**Table S6. Population comparison tests of the long/both variant(s)** **relationship of *timeless* with Tukey's HSD test*.*** List of statistically significant results of pairwise comparisons for different treatment groups (population, temperature and light). Abbreviations: Diff = mean difference between comparisons, Lwr = lower bound of 95% CI, Upr = upper bound of 95% CI, P adj = adjusted p-value, LL = constant light, LD = light-dark-cycle of 22:2. Populations are abbreviated as: Az = Azalea, Fa = Fairbanks, La = Lahti and Py = Pyhätunturi. Statistically significant values of treatment groups from the same continent are bolded. For all the pairwise comparisons, see Supplementary Table S7.

| **Treatment groups compared** | **Diff** | **Lwr** | **Upr** | **P adj** |
| --- | --- | --- | --- | --- |
| Fa_16_LL - Az_16_LD | 0.6045 | 0.0260 | 1.1830 | **0.0342*** |
| La_16_LD - Az_16_LD | 0.6859 | 0.0391 | 1.3326 | 0.0299 |
| Fa_16_LL - Az_19_LD | 0.5909 | 0.0124 | 1.1694 | **0.0417*** |
| La_16_LD - Az_19_LD | 0.6723 | 0.0255 | 1.3190 | 0.0358 |
| La_16_LL - Fa_16_LL | -0.6439 | -1.2224 | -0.0654 | 0.0189 |
| La_19_LL - Fa_16_LL | -0.6703 | -1.2488 | -0.0918 | 0.0125 |
| Py_16_LL - Fa_16_LL | -0.6361 | -1.2146 | -0.0576 | 0.0213 |
| Py_19_LL - Fa_16_LL | -0.6494 | -1.2279 | -0.0709 | 0.0173 |
| La_16_LL - La_16_LD | -0.7252 | -1.3720 | -0.0785 | **0.0175*** |
| La_19_LL - La_16_LD | -0.7517 | -1.3984 | -0.1049 | **0.0122*** |
| Py_16_LL - La_16_LD | -0.7175 | -1.3642 | -0.0707 | **0.0195*** |
| Py_19_LL - La_16_LD | -0.7308 | -1.3775 | -0.0840 | **0.0162*** |

Significance levels: * p<0.05, ** p<0.01, *** p<0.001.

**Table S7. Comparison of the different treatment groups in different populations with Tukey's HSD test.** Abbreviations Diff = mean difference between comparisons, Lwr = lower bound of 95% CI, Upr = upper bound of 95% CI, P adj = p-value. Populations are abbreviated as: Az = Azalea, Fa = Fairbanks, La = Lahti and Py = Pyhätunturi. Statistically significant p-values are bolded.

| **Treatment groups compared** | | | **Diff** | | **Lwr** | | **Upr** | **P adj** | | | |
| --- | --- | --- | --- | --- | --- | --- | --- | --- | --- | --- | --- |
| Az_16_LL - Az_16_LD | 0.3442 | | | -0.2343 | | 0.9227 | | 0.6610 | |  |  |
| Az_19_LD - Az_16_LD | 0.0136 | | | -0.5649 | | 0.5921 | | 1.0000 | |  |  |
| Az_19_LL - Az_16_LD | 0.1746 | | | -0.4039 | | 0.7530 | | 0.9979 | |  |  |
| Fa_16_LD - Az_16_LD | 0.3070 | | | -0.3397 | | 0.9538 | | 0.8983 | |  |  |
| Fa_16_LL - Az_16_LD | 0.6045 | | | 0.0260 | | 1.1830 | | **0.0342*** | |  |  |
| Fa_19_LD - Az_16_LD | 0.3287 | | | -0.3180 | | 0.9755 | | 0.8453 | |  |  |
| Fa_19_LL - Az_16_LD | 0.2930 | | | -0.2855 | | 0.8715 | | 0.8483 | |  |  |
| La_16_LD - Az_16_LD | 0.6859 | | | 0.0391 | | 1.3326 | | **0.0299*** | |  |  |
| La_16_LL - Az_16_LD | -0.0394 | | | -0.6178 | | 0.5391 | | 1.0000 | |  |  |
| La_19_LD - Az_16_LD | 0.4164 | | | -0.2304 | | 1.0632 | | 0.5435 | |  |  |
| La_19_LL - Az_16_LD | -0.0658 | | | -0.6443 | | 0.5127 | | 1.0000 | |  |  |
| Py_16_LD - Az_16_LD | 0.1989 | | | -0.4478 | | 0.8457 | | 0.9975 | |  |  |
| Py_16_LL - Az_16_LD | -0.0316 | | | -0.6101 | | 0.5469 | | 1.0000 | |  |  |
| Py_19_LD - Az_16_LD | 0.2163 | | | -0.4305 | | 0.8630 | | 0.9942 | |  |  |
| Py_19_LL - Az_16_LD | -0.0449 | | | -0.6234 | | 0.5336 | | 1.0000 | |  |  |
| Az_19_LD - Az_16_LL | -0.3306 | | | -0.9091 | | 0.2479 | | 0.7158 | |  |  |
| Az_19_LL - Az_16_LL | -0.1697 | | | -0.7481 | | 0.4088 | | 0.9985 | |  |  |
| Fa_16_LD - Az_16_LL | -0.0372 | | | -0.6840 | | 0.6096 | | 1.0000 | |  |  |
| Fa_16_LL - Az_16_LL | 0.2603 | | | -0.3182 | | 0.8388 | | 0.9291 | |  |  |
| Fa_19_LD - Az_16_LL | -0.0155 | | | -0.6623 | | 0.6313 | | 1.0000 | |  |  |
| Fa_19_LL - Az_16_LL | -0.0512 | | | -0.6297 | | 0.5273 | | 1.0000 | |  |  |
| La_16_LD - Az_16_LL | 0.3417 | | | -0.3051 | | 0.9884 | | 0.8080 | |  |  |
| La_16_LL - Az_16_LL | -0.3836 | | | -0.9621 | | 0.1949 | | 0.4976 | |  |  |
| La_19_LD - Az_16_LL | 0.0722 | | | -0.5746 | | 0.7189 | | 1.0000 | |  |  |
| La_19_LL - Az_16_LL | -0.4100 | | | -0.9885 | | 0.1685 | | 0.3944 | |  |  |
| Py_16_LD - Az_16_LL | -0.1453 | | | -0.7921 | | 0.5015 | | 0.9999 | |  |  |
| Py_16_LL - Az_16_LL | -0.3758 | | | -0.9543 | | 0.2027 | | 0.5296 | |  |  |
| Py_19_LD - Az_16_LL | -0.1280 | | | -0.7747 | | 0.5188 | | 1.0000 | |  |  |
| Py_19_LL - Az_16_LL | -0.3891 | | | -0.9676 | | 0.1894 | | 0.4752 | |  |  |
| Az_19_LL - Az_19_LD | 0.1610 | | | -0.4175 | | 0.7394 | | 0.9991 | |  |  |
| Fa_16_LD - Az_19_LD | 0.2934 | | | -0.3533 | | 0.9402 | | 0.9249 | |  |  |
| Fa_16_LL - Az_19_LD | 0.5909 | | | 0.0124 | | 1.1694 | | **0.0417*** | |  |  |
| Fa_19_LD - Az_19_LD | 0.3151 | | | -0.3316 | | 0.9619 | | 0.8799 | |  |  |
| Fa_19_LL - Az_19_LD | 0.2794 | | | -0.2991 | | 0.8579 | | 0.8863 | |  |  |
| La_16_LD - Az_19_LD | 0.6723 | | | 0.0255 | | 1.3190 | | **0.0358*** | |  |  |
| La_16_LL - Az_19_LD | -0.0530 | | | -0.6314 | | 0.5255 | | 1.0000 | |  |  |
| La_19_LD - Az_19_LD | 0.4028 | | | -0.2440 | | 1.0496 | | 0.5943 | |  |  |
| La_19_LL - Az_19_LD | -0.0794 | | | -0.6579 | | 0.4991 | | 1.0000 | |  |  |
| Py_16_LD - Az_19_LD | 0.1853 | | | -0.4614 | | 0.8321 | | 0.9988 | |  |  |
| Py_16_LL - Az_19_LD | -0.0452 | | | -0.6237 | | 0.5333 | | 1.0000 | |  |  |
| Py_19_LD - Az_19_LD | 0.2027 | | | -0.4441 | | 0.8494 | | 0.9969 | |  |  |
| Py_19_LL - Az_19_LD | -0.0585 | | | -0.6370 | | 0.5200 | | 1.0000 | |  |  |
| Fa_16_LD - Az_19_LL | | 0.1324 | | -0.5143 | | | 0.7792 | | 1.0000 |  |  |
| Fa_16_LL - Az_19_LL | | 0.4300 | | -0.1485 | | | 1.0084 | | 0.3243 |  |  |
| Fa_19_LD - Az_19_LL | | 0.1541 | | -0.4926 | | | 0.8009 | | 0.9999 |  |  |
| Fa_19_LL - Az_19_LL | | 0.1184 | | -0.4600 | | | 0.6969 | | 1.0000 |  |  |
| La_16_LD - Az_19_LL | | 0.5113 | | -0.1355 | | | 1.1581 | | 0.2416 |  |  |
| La_16_LL - Az_19_LL | | 0.2139 | | -0.7924 | | | 0.3645 | | 0.9851 |  |  |
| La_19_LD - Az_19_LL | | 0.2418 | | -0.4049 | | | 0.8886 | | 0.9836 |  |  |
| La_19_LL - Az_19_LL | | 0.2404 | | -0.8188 | | | 0.3381 | | 0.9607 |  |  |
| Py_16_LD - Az_19_LL | | 0.0244 | | -0.6224 | | | 0.6711 | | 1.0000 |  |  |
| Py_16_LL - Az_19_LL | | 0.2061 | | -0.7846 | | | 0.3723 | | 0.9894 |  |  |
| Py_19_LD - Az_19_LL | | 0.0417 | | -0.6051 | | | 0.6885 | | 1.0000 |  |  |
| Py_19_LL - Az_19_LL | | 0.2195 | | -0.7980 | | | 0.3590 | | 0.9814 |  |  |
| Fa_16_LL - Fa_16_LD | | 0.2975 | | -0.3493 | | | 0.9443 | | 0.9174 |  |  |
| Fa_19_LD - Fa_16_LD | | 0.0217 | | -0.6868 | | | 0.7302 | | 1.0000 |  |  |
| Fa_19_LL - Fa_16_LD | | 0.0140 | | -0.6608 | | | 0.6327 | | 1.0000 |  |  |
| La_16_LD - Fa_16_LD | | 0.3789 | | -0.3296 | | | 1.0874 | | 0.7951 |  |  |
| La_16_LL - Fa_16_LD | | 0.3464 | | -0.9931 | | | 0.3004 | | 0.7935 |  |  |
| La_19_LD - Fa_16_LD | | 0.1094 | | -0.5991 | | | 0.8179 | | 1.0000 |  |  |
| La_19_LL - Fa_16_LD | | 0.3728 | | -1.0196 | | | 0.2740 | | 0.7046 |  |  |
| Py_16_LD - Fa_16_LD | | 0.1081 | | -0.8166 | | | 0.6004 | | 1.0000 |  |  |
| Py_16_LL - Fa_16_LD | | 0.3386 | | -0.9854 | | | 0.3082 | | 0.8172 |  |  |
| Py_19_LD - Fa_16_LD | | 0.0907 | | -0.7992 | | | 0.6177 | | 1.0000 |  |  |
| Py_19_LL - Fa_16_LD | | 0.3519 | | -0.9987 | | | 0.2948 | | 0.7758 |  |  |
| Fa_19_LD - Fa_16_LL | | 0.2758 | | -0.9226 | | | 0.3710 | | 0.9521 |  |  |
| Fa_19_LL - Fa_16_LL | | 0.3115 | | -0.8900 | | | 0.2670 | | 0.7874 |  |  |
| La_16_LD - Fa_16_LL | | 0.0814 | | -0.5654 | | | 0.7281 | | 1.0000 |  |  |
| La_16_LL - Fa_16_LL | | 0.6439 | | -1.2224 | | | -0.0654 | | **0.0189*** |  |  |
| La_19_LD - Fa_16_LL | | 0.1881 | | -0.8349 | | | 0.4586 | | 0.9986 |  |  |
| La_19_LL - Fa_16_LL | | 0.6703 | | -1.2488 | | | -0.0918 | | **0.0125*** |  |  |
| Py_16_LD - Fa_16_LL | | 0.4056 | | -1.0524 | | | 0.2412 | | 0.5838 |  |  |
| Py_16_LL - Fa_16_LL | | 0.6361 | | -1.2146 | | | -0.0576 | | **0.0213*** |  |  |
| Py_19_LD - Fa_16_LL | | 0.3883 | | -1.0350 | | | 0.2585 | | 0.6484 |  |  |
| Py_19_LL - Fa_16_LL | | 0.6494 | | -1.2279 | | | -0.0709 | | **0.0173*** |  |  |
| Fa_19_LL - Fa_19_LD | | 0.0357 | | -0.6825 | | | 0.6110 | | 1.0000 |  |  |
| La_16_LD - Fa_19_LD | | 0.3572 | | -0.3513 | | | 1.0657 | | 0.8525 |  |  |
| La_16_LL - Fa_19_LD | | 0.3681 | | -1.0148 | | | 0.2787 | | 0.7213 |  |  |
| La_19_LD - Fa_19_LD | | 0.0877 | | -0.6208 | | | 0.7962 | | 1.0000 |  |  |
| La_19_LL - Fa_19_LD | | 0.3945 | | -1.0413 | | | 0.2523 | | 0.6253 |  |  |
| Py_16_LD - Fa_19_LD | | 0.1298 | | -0.8383 | | | 0.5787 | | 1.0000 |  |  |
| Py_16_LL - Fa_19_LD | | 0.3603 | | -1.0071 | | | 0.2865 | | 0.7481 |  |  |
| Py_19_LD - Fa_19_LD | | 0.1124 | | -0.8209 | | | 0.5960 | | 1.0000 |  |  |
| Py_19_LL - Fa_19_LD | | 0.3736 | | -1.0204 | | | 0.2731 | | 0.7017 |  |  |
| La_16_LD - Fa_19_LL | | 0.3929 | | -0.2539 | | | 1.0396 | | 0.6313 |  |  |
| La_16_LL - Fa_19_LL | | 0.3324 | | -0.9108 | | | 0.2461 | | 0.7089 |  |  |
| La_19_LD - Fa_19_LL | | 0.1234 | | -0.5234 | | | 0.7702 | | 1.0000 |  |  |
| La_19_LL - Fa_19_LL | | 0.3588 | | -0.9373 | | | 0.2197 | | 0.6005 |  |  |
| Py_16_LD - Fa_19_LL | | -0.0941 | | -0.7408 | | | 0.5527 | | 1.0000 | |  |
| Py_16_LL - Fa_19_LL | | -0.3246 | | -0.9031 | | | 0.2539 | | 0.7392 | |  |
| Py_19_LD - Fa_19_LL | | -0.0767 | | -0.7235 | | | 0.5700 | | 1.0000 | |  |
| Py_19_LL - Fa_19_LL | | -0.3379 | | -0.9164 | | | 0.2406 | | 0.6867 | |  |
| La_16_LL - La_16_LD | | -0.7252 | | -1.3720 | | | -0.0785 | | **0.0175*** | |  |
| La_19_LD - La_16_LD | | -0.2695 | | -0.9780 | | | 0.4390 | | 0.9810 | |  |
| La_19_LL - La_16_LD | | -0.7517 | | -1.3984 | | | -0.1049 | | **0.0122*** | |  |
| Py_16_LD - La_16_LD | | -0.4870 | | -1.1954 | | | 0.2215 | | 0.4416 | |  |
| Py_16_LL - La_16_LD | | -0.7175 | | -1.3642 | | | -0.0707 | | **0.0195*** | |  |
| Py_19_LD - La_16_LD | | -0.4696 | | -1.1781 | | | 0.2389 | | 0.4982 | |  |
| Py_19_LL - La_16_LD | | -0.7308 | | -1.3775 | | | -0.0840 | | **0.0162*** | |  |
| La_19_LD - La_16_LL | | 0.4558 | | -0.1910 | | | 1.1025 | | 0.4032 | |  |
| La_19_LL - La_16_LL | | -0.0264 | | -0.6049 | | | 0.5521 | | 1.0000 | |  |
| Py_16_LD - La_16_LL | | 0.2383 | | -0.4085 | | | 0.8850 | | 0.9856 | |  |
| Py_16_LL - La_16_LL | | 0.0078 | | -0.5707 | | | 0.5863 | | 1.0000 | |  |
| Py_19_LD - La_16_LL | | 0.2556 | | -0.3911 | | | 0.9024 | | 0.9737 | |  |
| Py_19_LL - La_16_LL | | -0.0055 | | -0.5840 | | | 0.5729 | | 1.0000 | |  |
| La_19_LL - La_19_LD | | -0.4822 | | -1.1289 | | | 0.1646 | | 0.3199 | |  |
| Py_16_LD - La_19_LD | | -0.2175 | | -0.9260 | | | 0.4910 | | 0.9975 | |  |
| Py_16_LL - La_19_LD | | -0.4480 | | -1.0947 | | | 0.1988 | | 0.4297 | |  |
| Py_19_LD - La_19_LD | | -0.2001 | | -0.9086 | | | 0.5084 | | 0.9990 | |  |
| Py_19_LL - La_19_LD | | -0.4613 | | -1.1081 | | | 0.1855 | | 0.3848 | |  |
| Py_16_LD - La_19_LL | | 0.2647 | | -0.3820 | | | 0.9115 | | 0.9651 | |  |
| Py_16_LL - La_19_LL | | 0.0342 | | -0.5443 | | | 0.6127 | | 1.0000 | |  |
| Py_19_LD - La_19_LL | | 0.2821 | | -0.3647 | | | 0.9288 | | 0.9434 | |  |
| Py_19_LL - La_19_LL | | 0.0209 | | -0.5576 | | | 0.5994 | | 1.0000 | |  |
| Py_16_LL - Py_16_LD | | -0.2305 | | -0.8773 | | | 0.4163 | | 0.9893 | |  |
| Py_19_LD - Py_16_LD | | 0.0173 | | -0.6911 | | | 0.7258 | | 1.0000 | |  |
| Py_19_LL - Py_16_LD | | -0.2438 | | -0.8906 | | | 0.4029 | | 0.9824 | |  |
| Py_19_LD - Py_16_LL | | 0.2478 | | -0.3989 | | | 0.8946 | | 0.9797 | |  |
| Py_19_LL - Py_16_LL | | -0.0133 | | -0.5918 | | | 0.5652 | | 1.0000 | |  |
| Py_19_LL - Py_19_LD | | -0.2612 | | -0.9079 | | | 0.3856 | | 0.9687 | |  |

Significance levels: * p<0.05, ** p<0.01, *** p<0.001.

**Table S8. Amount of daily light hours and temperature range per month from April to October in the study populations.** Temperatures are given as mean day/night temperatures of particular month.

|  |  | **April** |  | **May** |  | **June** |  | **July** |  | **August** |  | **September** |  | **October** |  |
| --- | --- | --- | --- | --- | --- | --- | --- | --- | --- | --- | --- | --- | --- | --- | --- |
| **Country** | **Population** | LD | °C | LD | °C | LD | °C | LD | °C | LD | °C | LD | °C | LD | °C |
| **Finland** | **Pyhätunturi** 67°00’N; 27°08’E  Altitude 178 m | 15.3 | 4/-7 | 19.2 | 10/0 | 24 | 17/7 | 21 | 20/10 | 16.7 | 17/7 | 12.8 | 10/2 | 9.1 | 3/-3 |
|  | **Lahti** 60°59’N;25°40’E  Altitude 82 m | 14.4 | 9/-1 | 17.0 | 16/3 | 18.7 | 20/8 | 17.9 | 23/11 | 15.5 | 21/10 | 12.6 | 15/5 | 9.9 | 8/1 |
| **USA** | **Fairbanks**, Alaska 64°55’N;147°59’W Altitude 180 m | 14.9 | 8/-6 | 18.2 | 17/3 | 20.7 | 23/9 | 19.5 | 24/11 | 16.2 | 20/8 | 12.8 | 14/2 | 9.4 | 1/-9 |
|  | **Azalea**, Oregon 42°48’N;123°13’W Altitude 498 m | 13.2 | 20/4 | 14.1 | 24/7 | 15.1 | 28/10 | 14.8 | 34/14 | 13.7 | 33/13 | 12.3 | 31/9 | 10.9 | 23/5 |

Data collected from http://astro.unl.edu/classaction/animations/coordsmotion/daylighthoursexplorer.html and from http://www.weatherbase.com.
